# Description of two new species of *Rissoella* Gray, 1847 (Mollusca, Gastropoda, Heterobranchia) from Venezuela, with a key to the Caribbean species  known for the genus

**DOI:** 10.3897/zookeys.115.1163

**Published:** 2011-07-05

**Authors:** Manuel Caballer, Jesus Ortea, Samuel Narciso

**Affiliations:** 1 Department of Oceanology and Coastal Sciences, Venezuelan Institute for Scientific Research, Carretera Panamericana km 11, Altos de Pipe, Miranda, Venezuela; 2 Department BOS, University of Oviedo, Calle Catedrático Valentín Andrés Álvarez s/n, 33006 Oviedo, Asturias, Spain; 3 FUDENA (Nature Defense Foundation), Calle Carabobo s/n, Chichiriviche, Falcón, Venezuela

**Keywords:** Rissoellidae, new species, *Thalassia*, *Dictyota*, Morrocoy, Isla de Aves, Southern Caribbean, Venezuela

## Abstract

Two new species of the genus *Rissoella* Gray, 1847 are described from Venezuela, one from the National Park Morrocoy, *Rissoella morrocoyensis* **sp. n.** and the other from the Wildlife Refuge Isla de Aves, *Rissoella venezolanicola* **sp. n.** *Rissoella morrocoyensis* **sp. n**. has a deep umbilicus (partly closed), preumbilical cord, black head, hypobranchial gland marked by a pale yellow boomerang-shaped ribbon and it lives on the leaves of the seagrass *Thalassia testudinum* Banks & König, 1805. *Rissoella venezolanicola* **sp. n.** has an angled preumbilical cord which extends to the columella delimiting a trapezoid, a hypobranchial gland marked by a yellow quaver-shaped ribbon and protoconch with fuchsia highlights. It lives on the brown alga *Dictyota* spp. The records of *Rissoella* in the Caribbean are revised and illustrations, a comparative table and a key to the Caribbean species known for the genus are provided.

## Introduction

The genus *Rissoella* Gray, 1847 consists of minute, less than 2 mm long gastropods, living on algae in shallow waters around the world. Their transparent shells have few distinguishing characters (Ponder and Yoo 1977), but the body exhibits pigmented organs that allow the separation of species (Ortea and Espinosa 2001; Ortea and Espinosa 2004; Rolán and Hernández 2004; Espinosa and Ortea 2009). Ponder and Yoo (1977) in the Pacific and Ortea and Espinosa (2004) in the Atlantic Ocean recognized a number of characteristic traits of the shell for identifying the different species using the proportions and the angle of the different whorls, the protoconch and especially the shape of the umbilicus, in addition to the colour of the body.

The natural history of *Rissoella* in western Atlantic was recently revised by Ortea and Espinosa (2004), who established 4 valid species for the area and described 7 new taxa from Cuba. Posteriorly, *Rissoella aliciae* Espinosa and Ortea, 2009 (Fig. 31), was added to the list. Nevertheless, the inventory of species is still incomplete. 10 species had been described in northern Caribbean, one in Central America (Tab. 1) and one in Brazil (*Rissoella ornata* Simone, 1995), but none in southern Caribbean, where only one species, *Rissoella caribaea* Rehder, 1943, has been recorded. The discovery of two members of the genus *Rissoella* with characters that differentiate them from other known species in the American Atlantic motivated this work.

## Material and methods

The specimens were collected by snorkelling in two localities of Venezuela; the National Park Morrocoy (March 2010) and the Wildlife Refuge Isla de Aves (August 2010). A Carl Zeiss stereomicroscope was used to take data on external anatomy and color patterns. The animals were photographed alive and preserved in ethanol 96 %. To prevent the progressive deterioration of the shells due to ethanol, the holotypes and some of the paratypes were kept dried.

To compare with other species of the genus, diagrams were made of shell, protoconch and umbilicus using an Olympus SZ16 stereomicroscope. A caliper was used to take measurements of each specimen. For other measurements such as the angle of the spire or umbilical angle, the methodology of Ortea and Espinosa (2004) was used.

SEM images were taken using a Hitachi S-2400 at the Central University of Venezuela. As umbilicus were partially closed by the expansion of the columellar edge, they were photographed (SEM) at an oblique angle (Figs 9–12) for better observation of preumbilical cord.

Abbreviations: SOM-IVIC, Marine Organisms Section of the Biological Collections of the Venezuelan Institute for Scientific Research (Register number 028), Miranda, Venezuela; FUDENA, Nature Defense Foundation, Falcon, Venezuela; PNM, National Park Morrocoy; RFSIA, Wildlife Refuge Isla de Aves.

## Systematics

### Family Rissoellidae Gray, 1850

#### Genus Rissoella

Gray, 1847

##### Type species:

*Rissoa? glaber* Alder (= *Rissoella glaber* (err. pro *glabra*) J. E. Gray, 1847; = *Rissoa? diaphana* Alder, 1848; = *Rissoa albella* Alder, 1844), by monotypy.

#### Rissoella morrocoyensis sp. n.

urn:lsid:zoobank.org:act:76349EA2-0BD1-49BE-B299-975357F50CA9

http://species-id.net/wiki/Rissoella_morrocoyensis

**Figures 1–8.**
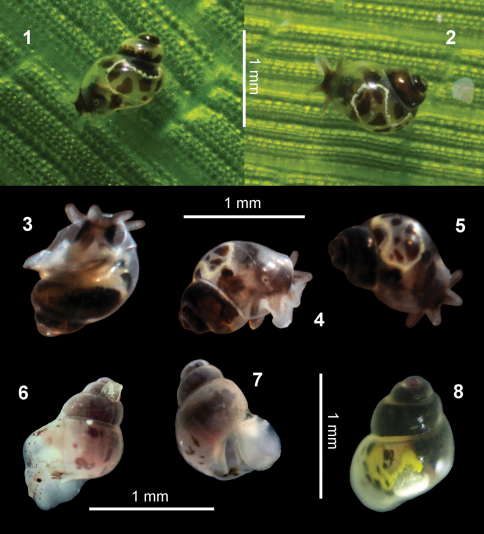
*Rissoella morrocoyensis* sp. n. **1–5**: on a leaf of *Thalassia testudinum* (**1–2**), ventral view (**3**), lateral view (**4**), dorsal view (**5**). *Rissoella venezolanicola* sp. n. **6–8**: lateral view (**6**), ventral view (**7**), dorsal view (**8**).

Figs 1–5 9–10 13–17 Tabs 1 2 4 

##### Description.

**Figures 9–12.**
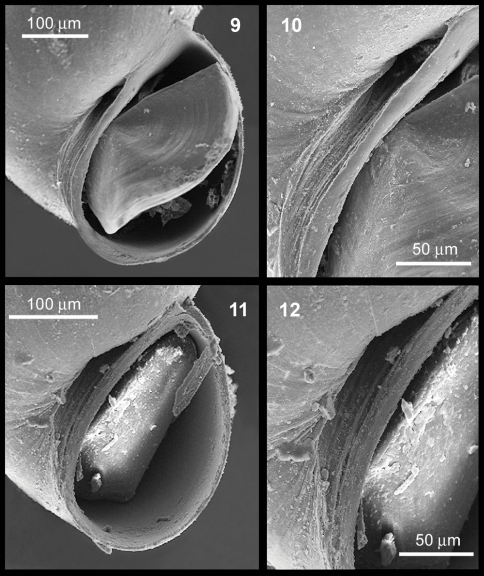
*Rissoella morrocoyensis* sp. n. **9–10**: view of the aperture and the umbilicus (**9**) detail of the umbilicus (**10**). *Rissoella venezolanicola* sp. n. **11–12**: view of the aperture and the umbilicus (**11**), detail of the umbilicus **12**.

**Figures 13–17.**
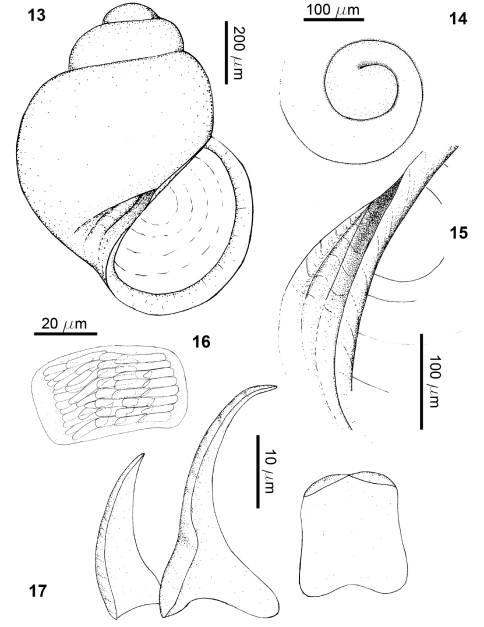
*Rissoella morrocoyensis* sp. n.: shell (**13**), protoconch (**14**), detail of the umbilicus (**15**), odontophoral cartilages (**16**), radular teeth (**17**).

Shell very small (Tab. 2), smooth, translucent and fragile (Fig. 13); protoconch about half a whorl after the nucleus (Fig. 14); teleoconch of two and a half whorls to two and three quarters, convex profile, well marked suture; aperture semicircular, columella almost straight; umbilicus small, very narrow and deep, partially closed by the expansion of the columellar edge, with a preumbilical cord (Figs 9–10, 15), average height of the last whorl, 85% of shell length; average height of the aperture, 60% of shell length (Tab. 2); shell length/width ratio = 1.32; spiral angle = 60°; umbilical angle = 21°–28°.

**Table 1.** Valid species of the genus *Rissoella* Gray, 1847 in the Caribbean.

Species	Type locality
*Rissoella caribaea* Rehder, 1943	USA
*Rissoella galba* Robertson, 1961	Bahamas
*Rissoella gandocaensis* Ortea & Espinosa, 2001	Costa Rica
*Rissoella ameliae* Ortea & Espinosa, 2004	Cuba
*Rissoella belkisae* Ortea & Espinosa, 2004	Cuba
*Rissoella dianae* Ortea & Espinosa, 2004	Cuba
*Rissoella zaidae* Ortea & Espinosa, 2004	Cuba
*Rissoella florae* Ortea & Espinosa, 2004	Cuba
*Rissoella elsae* Ortea & Espinosa, 2004	Cuba
*Rissoella taniae* Ortea & Espinosa, 2004	Cuba
*Rissoella aliciae* Espinosa & Ortea, 2009	Cuba
*Rissoella morrocoyensis* sp. n.	Venezuela
*Rissoella venezolanicola* sp. n.	Venezuela

Operculum semicircular, translucent amber, rather opaque in the center, 453 µm long by 255 µm wide in a shell of 0.92 mm length; inner side with a triangular projection with spearhead-shaped apex.

Head dark brown to black; black eyes set in a circular area of translucent white colour; oral lobes wider and shorter than the cephalic tentacles; both translucent, dark brown or black, completely or just at the base (Figs 1–2); foot slightly bilobed, with a white hyaline sole; dorsal part of the foot, white or with an irregular dark drawing (Fig. 3); flanks of the animal the same colour as the head (Fig. 4); mantle white, with several black blotches around the hypobranchial gland; hypobranchial gland translucent white, with white spots,  irregular black blotches and a pale yellow band marking its boomerang-shaped contour with no transversal bands (Fig. 5); visceral mass in the first whorls, dark brown to black.

Odontophoral cartilages rectangular, with polygonal uncini of 10–16 µm long, regularly imbricated (Fig. 16); radular formula of a specimen 1.02 mm shell length, 15 × 1.1.R.1.1; rachidian tooth wide, with bilobed apex forming two smooth cusps (Fig. 17), slightly shorter than marginal tooth; lateral tooth thorn-shaped, 34.2 µm long, with the apex hooked and smooth, imbricated with the opposite tooth above the rachidian; marginal tooth triangular, 26.1 µm long, with sharp apex.

##### Types.

Holotype, 1.06 mm × 0.82 mm, collected alive (June 10, 2010), preserved dry and deposited in SOM-IVIC (IVICCMT005). Paratypes 1–2, 1.25 mm × 0.86 mm and 1 mm × 0.69 mm, preserved in etanol 96%, SOM-IVIC (IVICCMT006). Paratypes 3–4, 1.16 mm × 0.80 mm and 1.06 mm × 0.82 mm, preserved dry, SOM-IVIC (IVICCMT007). Paratypes 5–7, 0.90 mm × 0.68 mm; 0.86 mm × 0.78 mm and 1.10 mm × 0.80 mm, preserved dry, FUDENA (CFPM0001).

##### Further material.

1.02 mm × 0.86 mm, collected (March 24, 2010) in Boca Grande, PNM (10°51'01.71"N, 68°14'16.48"W), used to obtain the radula, therefore the specimen was destroyed.

##### Type locality.

Boca Grande, National Park Morrocoy, Venezuela (10°51'28.85"N, 68°13'17.04"W), at the base of the leaves of *Thalassia testudinum*, 1 m depth.

##### Etymology.

*morrocoyensis*, latinization of *morrocoy*, place name of National Park Morrocoy, Venezuela, where the type locality is located.

##### Remarks.

According to the classification given by Ortea and Espinosa (2004), *Rissoella morrocoyensis* sp. n. would cluster within the Caribbean species group with a preumbilical cord, which include: *Rissoella zaidae* Ortea & Espinosa, 2004 (Fig. 27), *Rissoella florae* Ortea & Espinosa, 2004 (Fig. 28), *Rissoella elsae* Ortea & Espinosa, 2004 (Fig. 29) and *Rissoella taniae* Ortea & Espinosa, 2004 (Fig. 30). *Rissoella morrocoyensis* sp. n. has a preumbilical cord thicker than these four species, all of which have their type locality on the shores of Cuba. Additionally, the body colouration of *Rissoella florae*, *Rissoella elsae* and *Rissoella taniae* is very different as well as the shape and proportions of their shells, which are larger.

The shell of *Rissoella zaidae* is similar in size to that of *Rissoella morrocoyensis* sp. n., but the animal has a different colour and the oral palps equal the cephalic tentacles, while in *Rissoella morrocoyensis* sp. n., they are shorter. Additionally, *Rissoella zaidae* has transverse yellow bands in the hypobranchial gland.

*Rissoella contrerasi* Rolán & Hernandez, 2004, from Africa, has the design on the hypobranchial gland similar to *Rissoella morrocoyensis* sp. n., but it distinguishes by the curved inner edge of the columella, by the oral lobes being longer than the cephalic tentacles, and by it’s different body colour (black), which is displayed even on the sole of the foot and on the hypobranchial gland. In addition, the whorls of the shell and the aperture are different and it has a wider umbilicus.

**Table 2.** *Rissoella morrocoyensis* sp. n. Measurements of specimens. (%) means percentage compared to the total length of the shell.

	Protoconch (whorls)	Last whorl(mm)	Aperture(mm)	Length /width
Holotype				
1.06 × 0.82 mm	1.5	0.92 (87%)	0.64 (60%)	1.29
Paratypes				
1–1.25 × 0.86 mm	—	—	—	1.45
2–1 × 0.69 mm	—	—	—	1.44
3–1.16 × 0.80 mm	1.25	0.92 (79%)	0.70 (60%)	1.45
4–1.06 × 0.82 mm	1.15	0.90 (85%)	0.64 (60%)	1.29
5–0.90 × 0.68 mm	1	0.80 (89%)	0.54 (60%)	1.32
6–0.86 × 0.78 mm	1.5	0.76 (88%)	0.58 (67%)	1.10
7–1.10 × 0.80 mm	1.25	0.92 (84%)	0.66 (60%)	1.37
Further material				
1.02 × 0.86 mm	1	0.85 (83%)	0.57 (56%)	1.18
0.92 × 0.70 mm	—	—	0.46	1.31
Average		85%	60%	1.32

#### Rissoella venezolanicola sp. n.

urn:lsid:zoobank.org:act:69FA6D90-9365-4ADD-92DA-820BC27EC917

http://species-id.net/wiki/Rissoella_venezolanicola

Figs 6–8 11–12 18–22 Tabs 1 3 4 

##### Description.

**Figures 18–22.**
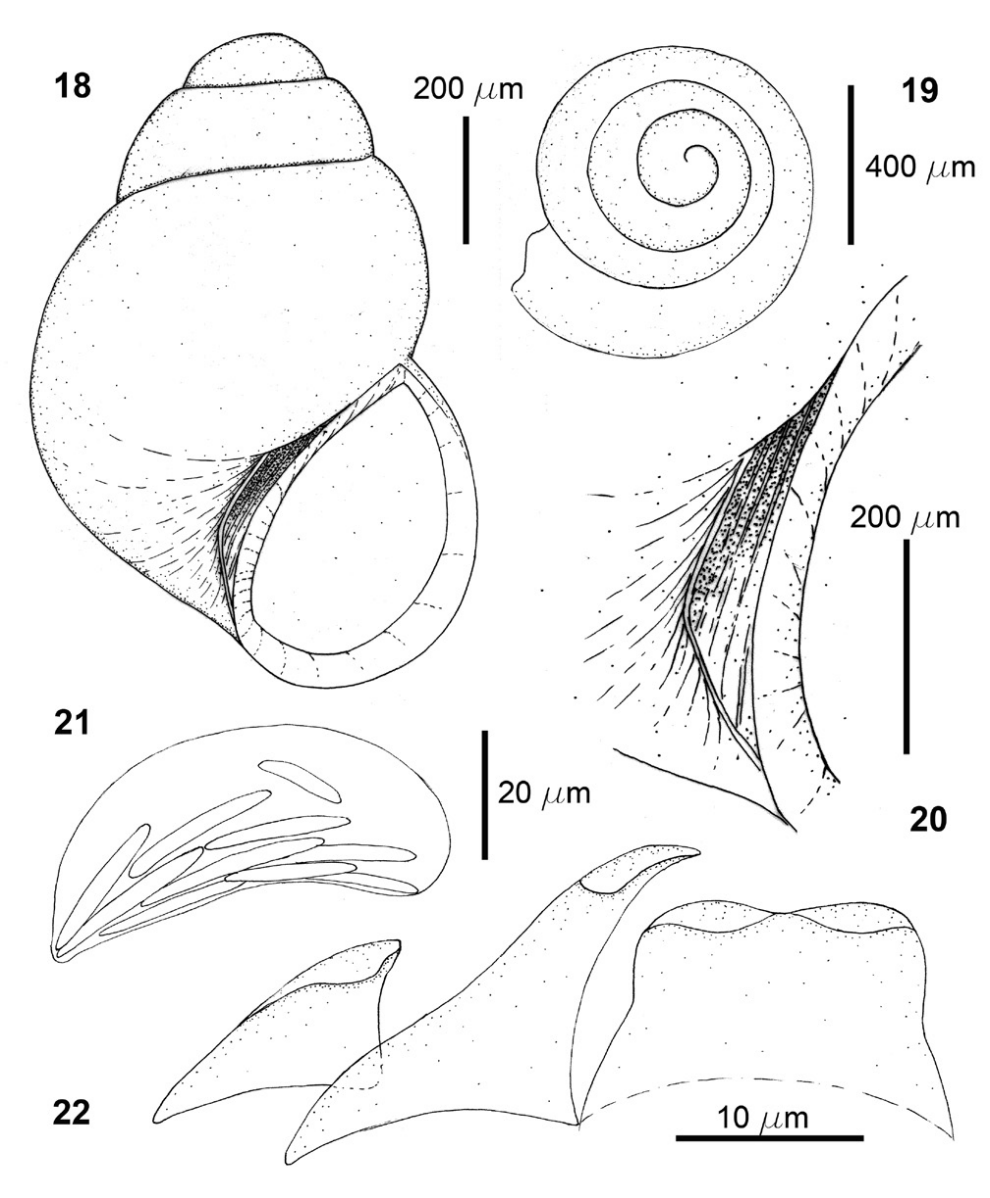
*Rissoella venezolanicola* sp. n.: shell (**18**), protoconch (**19**), detail of the umbilicus (**20**), odontophoral cartilages (**21**), radular teeth (**22**).

Shell very small (Tab. 3), smooth and translucent (Fig. 18); protoconch about half a whorl after the nucleus (Fig. 19); teleoconch of two whorls and three quarters; aperture oval, with the columella slightly bowed and arched; umbilicus narrow and deep, slightly closed by the expansion of the columellar edge; preumbilical cord extended, with an angle delimiting and closing the umbilicus forming a trapezoid (Figs 11–12, 20); average height of the last whorl, 82.9% of shell length; average height of the aperture, 49.6% of shell length (Tab. 3); Shell length/width ratio = 1.52; spiral angle = 58°; umbilical angle = 23°-25°.

**Figures 23–31.**
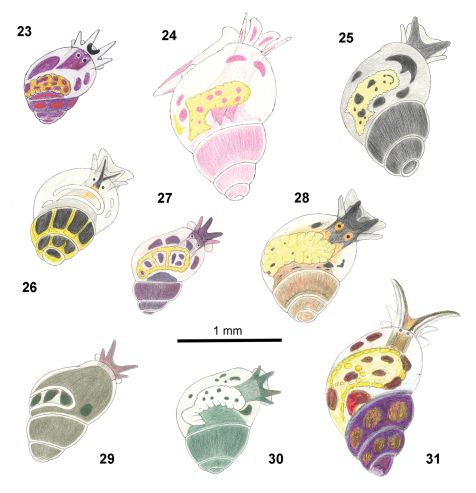
Color illustrations adapted from the original descriptions (Ortea and Espinosa 2001; 2004; Espinosa and Ortea 2009). *Rissoella gandocaensis* (**23**), *Rissoella ameliae* (**24**), *Rissoella belkisae* (**25**), *Rissoella dianae* (**26**), *Rissoella zaidae* (**27**), *Rissoella florae* (**28**), *Rissoella elsae* (**29**), *Rissoella taniae* (**30**), *Rissoella aliciae* (**31**).

Operculum oval, translucent with an amber tinge, membranous consistence; head, flanks, oral lobes and cephalic tentacles translucent white with scattered black to greenish brown dots (Fig. 6); eyes black; foot translucent white (Fig. 7); hypobranchial gland translucent white with scattered blotches black to greenish brown and a distinctive yellow design quaver-shaped (Fig. 8); visceral mass in the first whorls, black; protoconch with fuchsia highlights in live animals.

Odontophoral cartilages kidney-shaped, with large uncini placed longitudinally (Fig. 21); radular formula of an specimen 0.96 mm shell length, 16 × 1.1.R.1.1; rachidian tooth wide, with bilobed apex forming two smooth cusps (Fig. 22), slightly larger than marginal tooth; lateral tooth triangular, 28.3 µm long, with the apex hooked and smooth; imbricated with the opposite tooth above the rachidian; marginal tooth triangular and curved, 16.3 µm long, with blunt apex.

**Table:** **Table 3.** *Rissoella venezolanicola* sp. n. Measurements of specimens. (%) means percentage compared to the total length of the shell.

	Protoconch (whorls)	Last whorl (mm)	Aperture(mm)	Length /width
Holotype				
1.02 × 0.66 mm	1.25	0.82 (80%)	0.46 (45%)	1.54
Paratypes				
0.96 × 0.64 mm	1.25	0.80 (83%)	0.52 (54%)	1.50
0.54 × 0.46 mm	1.5	0.46 (85%)	0.38 (70%)	1.18
Average		83%	*50%/57%	*1.52/1.4

* Excluding the juvenile specimen.

**Table 4.** Synthesis of characters of the Caribbean species of *Rissoella* Gray, 1847 based on Robertson (1961), Wise (1998), Ortea and Espinosa (2001; 2004), Espinosa and Ortea (2009). Aa = Average height of the aperture vs shell length, Al = Average height of the last whorl vs shell length, E = Eyes, H = Habitat, HG = Hypobranchial gland, LW = Shell length/width ratio, Pc = Preumbilical cord, Pf = Pigmentation of the body in the firsts whorls, Rf = Radular formula, Rt = lateral and marginal teeth, Sa = Spiral angle, U = Umbilicus, Ua = Umbilical angle.

	Shell	Al	Aa	LW	Sa	Ua	U	Pc	E	HG	Band around HG	Pf	Rf	Rt	H
*Rissoella caribaea*	TransparentTo 1.8 mm length	75–80%	50%	1.42	60°	25°	Narrow with a keel. Partly closed by columellar edge	Absent	Black on unpigmented areas	Grey with black blotches	Pale yellow. Boomerang-shaped with transversal bands	Homogeneous black	30 × 1.1.R.1.1.	Slightly serrated	Clean rocks and coral rubleRed and green algae on mangrove rootsShallow
*Rissoella galba*	Transparent with white band around umbilical regionTo 0.68 mm length	-	-	-	-	-	Narrow	Absent	Black	Pale yellow	Absent	Homogeneous pale brown	-	-	Filamentous green algae on rocks or filamentous red algae on mangrove rootsIntertidal
*Rissoella gandocaensis*	TransparentTo 0.8 mm length	-	-	-	-	-	With a slight keel	Absent	Black on clear areas	Yellow with violet blotches	Absent	Violet with red blotches	-	-	-
*Rissoella ameliae*	TransparentTo 1.62 mm length	75%	50%	1.57	45°	18°	Narrow and deep	Absent	Unpigmented or absent	Yellow with pale violet blotches	Absent	Homogeneous pale violet	-	-	Rocks and coral ruble on sand in the coral slope15–18 m deep
*Rissoella belkisae*	TransparentTo 1.2 mm length	80%	50%	1.38	60°	25°	Narrow but not deep	Absent	Unpigmented or absent	Yellow with black blotches	Absent	Homogeneous dark grey	-	-	Rocks and coral ruble on sand in the coral slope20–27 m deep
*Rissoella dianae*	TransparentTo 1.15 mm length	75%	44%	1.6	40°	30°	Wide and deep. Partly closed by columellar edge	Absent	Black	Translucent	Curved, Yellowish white, broken at the back	Brilliant Yellow with big square-shaped black blotches	-	-	Rocks and coral ruble on sand in the coral slope25–30 m deep
*Rissoella zaidae*	TransparentTo 1 mm length	78%	50%	1.63	50°	30°	Narrow and deep	Present	Black on unpigmented areas	Kidney-shaped, pale violet with lilac blotches	Gold-yellow with transverse bands	Homogeneous lilac	-	-	Rocks and coral ruble on sand in the coral slope15–18 m deep
*Rissoella florae*	TransparentTo 1.2 mm length	79%	55%	1.35	60°	18°	Narrow and deep	Present	Black on lighter areas of orange hue	Intense yellow with black spots	Absent	Homogeneous brown-grey	-	-	Rocks and coral ruble on sand in the coral slope25–30 m deep
*Rissoella elsae*	TransparentTo 1.2 mm length	76%	50%	1.69	45°	25°	Nearly closed by columellar edge	Present	Unpigmented or absent	White with big green blotches	Kidney-shaped, white without transverse bands	Homogeneous green	-	-	Rocks and coral ruble on sand in the coral slope15–25 m deep
*Rissoella taniae*	TransparentTo 1.1 mm length	82%	52%	1.4	65°	43°	Wide	Present	Unpigmented or absent	Curved. White with big green spots	Absent	Homogeneous dark green	-	-	Rocks and coral ruble on sand in the slope15 m deep
*Rissoella aliciae*	TransparentTo 1.45 mm length	67%	40%	1.52	45°	22°	Wide and deep	Absent	Black on lighter areas	Pale yellow with black blotches	Yellow. Discontinuous, broken at the back	Violet with circular brown blotches	-	-	Filamentous algae on rocks in sandy bottoms35 m deep
*Rissoella morrocoyensis* sp. n.	Transparent To 1.25 mm length	85%	60%	1.32	60°	21°–28°	Very narrow and deep. Partly closed by columellar edge	Present	Black on unpigmented areas	Translucent white, with black blotches	Pale yellow. Boomerang-shaped with no transversal bands	Homogeneous black	15 x 1.1.R.1.1.	Smooth	Leaves of Thalassia near mangroves1 m deep
*Rissoella venezolanicola* sp. n.	Transparent To 1.02 mm	83%	57%	1.52	58°	23°–25°	Narrow and deep	Long, with an angle, forming a trapezoid	Black on unpigmented areas	Translucent white with black to greenish brown blotches	Yellow. Quaver-shaped	Homogeneous black	16 x 1.1.R.1.1.	Smooth	Coral reef on on Dictyota spp.10 m deep

##### Types.

Holotype, 1.02 µm × 0.66 µm, collected alive (August 2, 2010), preserved dry and deposited in FUDENA (CFRFSIA0002). Paratype 1, 0.96 × 0.64 µm, SOM-IVIC (IVICCMT008), used to get the radula, shell preserved dry. Paratype 2, 0.54 × 0.46 µm, preserved dry, SOM-IVIC (IVICCMT009).

##### Type locality.

Leeward patch reef, Isla de Aves, Venezuela (15°39'54.2"N, 63°37'17.6"W), on *Dictyota* spp., 10 m depth.

##### Further localities.

Paratypes: Northern end, Isla de Aves, Venezuela, (15°40'24.7"N, 63°37'11"W), on *Dictyota* spp., 10 m depth.

##### Etymology.

*venezolanicola* latinization of *venezolana*, inhabitant of Venezuela.

##### Remarks.

Due to the presence of the preumbilical cord, *Rissoella venezolanicola* sp. n., is comparable to *Rissoella zaidae*, *Rissoella florae*, *Rissoella elsae*, *Rissoella taniae* (Ortea and Espinosa 2004) (listed above) and *Rissoella morrocoyensis* sp. n. But it is different because in none of them the preumbilical cord delimits and closes the umbilicus, neither the hypobranchial gland has a yellow quaver-shaped design. Additionally, *Rissoella venezolanicola* sp. n. differs from all these species by:

– *Rissoella zaidae*: (shell length/width ratio = 1.63, spiral angle = 50°, umbilical angle = 30°) the whorls of the spire are more angled and tilted, the umbilicus is in the middle of the last whorl, the head, oral lobes, cephalic tentacles and the visceral mass in the first whorls are lilac and the hypobranchial gland is kidney-shaped, pale violet with lilac blotches and bounded by a yellow ribbon with transverse bands.

– *Rissoella florae*: (shell length/width ratio = 1.35, umbilical angle = 18°) the head and oral lobes are black, the cephalic tentacles are different in colour than the oral lobes, the eyes are located in lighter areas of orange hue, the visceral mass in the first whorls is brown or orange and the hypobranchial gland is lemon-yellow without any design on it.

– *Rissoella elsae*: (shell length/width ratio = 1.69, spiral angle = 45°) the shell is more conical, the preumbilical cord is higher than the columellar wall, the head, oral lobes and cephalic tentacles are lilac, the mantle is green and hides the eyes, and the hypobranchial gland has large patches of dark green and is enclosed by a white ribbon with transverse bands.

– *Rissoella taniae*: (shell length/width ratio = 1.4, spiral angle = 65°, umbilical angle = 43°) the shell is spherical with slightly globose whorls, the umbilicus is very open, the head, oral lobes, cephalic tentacles and visceral mass in the first whorls are dark green, there are no eyes apparently and the hypobranchial gland is white with green spots.

– *Rissoella morrocoyensis* sp. n.: (shell length/width ratio = 1.32) the umbilicus is deeper and more closed by the expansion of the columellar edge and the head, oral lobes and cephalic tentacles are black.

Two species from Africa have a design on the hypobranchial gland similar to *Rissoella venezolanicola* sp. n.: *Rissoella luteonigra* Rolán & Rubio, 2001 and *Rissoella trigoi* Rolán & Hernández, 2004. *Rissoella venezolanicola* sp. n. differs from these species in:

– *Rissoella luteonigra*;is bigger (1.8 mm), with a narrower and longer shell, lacking preumbilical cord. Animals are black homogeneous (Rolán and Rubio 2001).

– *Rissoella trigoi*; has a rough protoconch with cavities in the suture, the preumbilical cord parallel to the expansion of the columellar edge until it converges (divergent in *Rissoella venezolanicola* sp. n.) and the animals are black with a white drawing in the hypobranchial gland (Rolán and Hernández 2004), very simple and very different.

## Discussion

The species of the family Rissoellidae are difficult to study and to identify, because their small transparent shells have only a few characters. Thus, identification should also be based on the color patterns of the animals facilitating correct recognition (Sasaki 2008). The lack of consensus on the correct characters to segregate species could explain why it is one of the least studied families of micromolluscs (Okutani 2000). For example in Japan, which is a country with a long malacological tradition, there are at least 10 morphospecies known, but most of them yet undescribed (Hasegawa 2000).

Species from this family belonging to the genus *Rissoella* have low dispersal abilities because of the absence of a pelagic larval stage. They are usually found in very specific habitats (Ortea and Espinosa 2004) and they tend to be microendemic, although their small size could facilitate passive transport in floating elements covered by algae. Therefore, their distribution should be established solely on the basis of publications that provide sufficient data for unambiguous identification. Concluding from this, most of the historical records of *Rissoella* species in Western Atlantic have to be considered doubtful.

In the Caribbean Sea, *Rissoella caribaea* is the most controversial species with the widest known distribution (Ortea and Espinosa 2004). The original description of *Rissoella caribaea* by Rehder (1943) did not allow to distinguish it from other species of the genus in the West Atlantic, but Wise (1998) compared his specimens from Florida with the holotype of this species (USNM No. 536046) and published a detailed redescription. According to him, *Rissoella caribaea* has a deep and narrow umbilicus without preumbilical cord, several strands or streaks of uniform height leaving from the umbilicus, a characteristic and prominent keel, bifid nose, gray-black body and hypobranchial gland, the last surrounded by a yellow ribbon with transverse bands, and a radula with 30 rows of serrated teeth. Although there is some theoretical geographic overlap, these characters clearly separate it from *Rissoella morrocoyensis* sp. n. and *Rissoella venezolanicola* sp. n.

*Rissoella caribaea* has been cited from:

–	Florida (type locality).

–	Florida, Bahama and Puerto Rico by Robertson (1961), who interprets the large differences in shell, body color and habitat along its distribution as intraspecific variation.

–	Puerto Rico by Ortiz-Corps (1985) just as a compilation of references.

–	Florida to Puerto Rico by Abbott (1974), with a description and figures.

–	Curaçao by Jong and Coomans (1988), based on the species description given by Abbott (1974).

–	Mexico by Vokes and Vokes (1983), with a black and white photograph that doesn’t permit to distinguish this species.

–	Northern Brazil, where Rios (1994) reproduced the same image that appeared in Abbott’s book (1974).

–	Bahamas by Redfern (2001), with images of the shell and the live animal. Curiously, the shell shown lacks the characteristic keel of the species shown by Wise (1998).

–	Nicaragua by Rolán and Hernández (2004) in the legend of an illustration.

–	Mexico by Felder and Camp (2009), on a checklist based on Vokes and Vokes (1983), Wise (1998) and Hicks et al. (2001).

–	Venezuela by Bitter et al. (2009), in a table without anatomic confirmation nor supply of images of the shell or the animal body.

–	Puerto Rico, Mexico, Belize and Aruba-Bonaire-Curaçao by Miloslavich et al. (2010) in a checklist of previous records.

Most of these authors repeat previous records (mainly Abbott’s), but very few of them give anatomical data or useful images to distinguish the species. Therefore, we consider it more likely that the real distribution of *Rissoella caribaea* is that given by Abbott (1974), from Florida to Puerto Rico. All other records require anatomical confirmation.

The record of Bitter et al. (2009) probably refers to *Rissoella morrocoyensis* sp. n. because of the proximity to its type locality.

*Rissoella galba* (Tab. 4) is a very rare and characteristic species (Robertson 1961), which has been cited from:

–	Bahama Islands (type locality) in low abundance cohabiting with hundreds of *Rissoella caribaea* (Robertson 1961).

–	Cozumel Island (Mexico) by Moore (1973), from shells in sediments, on a checklist without data or images.

–	Puerto Rico by Ortiz-Corps (1985) just as a compilation of references.

–	Bahamas (Abaco) by Redfern (2001), who only got a few empty shells in sediments from 10 to 23 m depth.

Abbott (1974) considers this species endemic for the Bahama Islands, we agree with this statement. Specimens from Mexico and Puerto Rico possibly refer to a related but undescribed species.

*Rissoella ornata* has been recorded from the coasts of Yucatan (Mexico) by Rolán and Hernández (2004), but their specimens are different from the species of Simone (1995) (Ortea and Espinosa 2004). So, the record in the Caribbean for *Rissoella ornata*, whose type locality is in southern Brazil, is considered to represent a misindentification.

None of the species of *Rissoella* described by Ortea and Espinosa (2001; 2004) and Espinosa and Ortea (2009) has been recorded from outside Cuba or Costa Rica, respectively. A synthesis of their main characters based on the original descriptions is shown in Figs 23–31 and Table 4.

This is the second record of species of the genus *Rissoella* for Venezuela and the mainland of the southern Caribbean, the first confirmed by anatomical characters. Thus, the the list of valid species in the Caribbean is now raised to 13 (Tab. 1). Even when the number of species of the genus in the Caribbean is likely to increase with future targeted searches and the exploration of new areas, a key is provided to allow the user to distinguish whether their specimens are already described or not.

In conclusion, we suggest that information on additional characters should be supplied when describing and characterising species of the genus *Rissoella* in the Caribbean. At least, detailed descriptions of the umbilicus (preumbilical cord, keel) displayed by line drawings or SEM photos, and colour illustrations of the living animals (including observations on the presence/absence and shape of the band around the hypobranchial gland) are urgently needed for an unambiguous identification.

### Key to the Rissoella species known from the Caribbean

**Table:** 

1	Shell with wide umbilicus. No preumbilical cord	2
–	Shell with preumbilical cord in the umbilicus	8
2	Gonad and digestive gland of uniform colouration in the first whorls	3
–	Gonad and digestive gland with different colouration in the first whorls	6
3	Shell with an opaque white spiral band extending around de umbilical region	*Rissoella galba*
–	Shell lacking a white band around de umbilical region	4
4	Hypobranchial gland surrounded by a yellow ribbon kidney-shaped, with transverse bands	*Rissoella caribaea*
–	Hypobranchial gland not surrounded by a ribbon	5
5	Oral lobes same color than the cephalic tentacles. Hypobranchial gland yellow with pink/violet blotches. Pink head	*Rissoella ameliae*
–	Oral lobes different in color than the cephalic tentacles. Hypobranchial gland yellow with black blotches. Dark grey or black head	*Rissoella belkisae*
6	Hypobranchial gland not conspicuous, surrounded by a discontinuous pale band, interrupted around the perimeter. White operculum	*Rissoella dianae*
–	Hypobranchial gland conspicuous and yellow	7
7	Hypobranchial gland not surrounded by a ribbon	*Rissoella gandocaensis*
–	Hypobranchial gland surrounded by a yellow ribbon kidney-shaped, without transverse bands	*Rissoella aliciae*
8	Eyes lacking or not visible	9
–	Eyes present and visible	10
9	Hypobranchial gland white with green blotches, surrounded by a white ribbon kidney-shaped, without transverse bands. Lilac head	*Rissoella elsae*
–	Hypobranchial gland white, not surrounded by a ribbon. Green head	*Rissoella tainae*
10	Hypobranchial gland surrounded by a band or with a design	11
–	Hypobranchial gland intense yellow, not surrounded by a ribbon. Eyes in lighter areas of orange hue	*Rissoella florae*
11	Hypobranchial gland translucent white, surrounded by a yellow quaver-shaped ribbon	*Rissoella venezolanicola*
–	Hypobranchial gland surrounded by a kidney-shaped ribbon	12
12	Hypobranchial gland violet with lilac blotches, surrounded by a gold-yellow band with transverse bands	*Rissoella zaidae*
–	Hypobranchial gland translucent white, surrounded by a pale yellow band without transverse bands	*Rissoella morrocoyensis*

## Supplementary Material

XML Treatment for Rissoella

XML Treatment for Rissoella morrocoyensis

XML Treatment for Rissoella venezolanicola
